# Infiltrating cells from host brain restore the microglial population in grafted cortical tissue

**DOI:** 10.1038/srep33080

**Published:** 2016-09-12

**Authors:** Cong Wang, Sijue Tao, Yukun Fang, Jing Guo, Lirui Zhu, Shengxiang Zhang

**Affiliations:** 1 Gansu Key Laboratory of Biomonitoring and Bioremediation for Environmental Pollution, School of Life Sciences, Lanzhou University, Lanzhou 730000, China

## Abstract

Transplantation of embryonic cortical tissue is considered as a promising therapy for brain injury. Grafted neurons can reestablish neuronal network and improve cortical function of the host brain. Microglia is a key player in regulating neuronal survival and plasticity, but its activation and dynamics in grafted cortical tissue remain unknown. Using two-photon intravital imaging and parabiotic model, here we investigated the proliferation and source of microglia in the donor region by transplanting embryonic cortical tissue into adult cortex. Live imaging showed that the endogenous microglia of the grafted tissue were rapidly lost after transplantation. Instead, host-derived microglia infiltrated and colonized the graft. Parabiotic model suggested that the main source of infiltrating cells is the parenchyma of the host brain. Colonized microglia proliferated and experienced an extensive morphological transition and eventually differentiated into resting ramified morphology. Collectively, these results demonstrated that donor tissue has little contribution to the activated microglia and host brain controls the microglial population in the graft.

The adult brain has a limited capacity of self-repairing after neuronal loss caused by trauma or disease. During last three decades, many experimental studies have explored the possibility to restore the lost functions by replacing damaged brain area with embryonic neural tissues. These studies have achieved remarkable results in showing that grafted neurons can successfully survive and synaptic connections can be established between the host and donor cells^1^,^2^,^3^. Importantly, grafted neurons are electrophysiologically active in responding to adjacent host cortex^4^,^5^,^6^, and the lost functions can be partially recovered after transplantation^7^,^8^,^9^.

For a successful transplantation, the entire cell populations of cortical tissue including neurons and different subsets of glial cells have to be reestablished. However, previous studies mostly focused on the survival and development of grafted neurons, and little information was available regarding the other essential cell subsets in cortical tissue. One of the key cell subsets that may affect the survival of graft tissue and reestablishment of synaptic connections is microglia. As the immunocompetent cells in the central nervous system (CNS)^10^,^11^,^12^, microglia show remarkable functional diversity under different conditions in brain. During early development, yolk sac-derived microglia enter the developing CNS and distribute widely in the brain^13^,^14^,^15^. Colonized microglia actively participate in regulating the number of neural precursor cells and wiring the axonal input^16^,^17^. In the adult brain, the number of microglia remains relatively stable but microglial processes are highly motile^18^. Resting microglia are involved in the surveillance and support of the CNS microenvironment^19^,^20^,^21^. In addition, they also contribute to synapse elimination and learning-dependent synapse formation^22^,^23^. Under pathological conditions, microglia are rapidly activated and play vital roles in immunoreactions and CNS pathologies^20^,^24^,^25^. Activated microglia can derive from different sources in different neuropathological conditions in adult brain^26^,^27^,^28^,^29^. The dynamics and sources of microglia are well described in healthy and diseased brain, but it is still undefined about the microglial activation and population dynamics in grafted embryonic tissue.

In this study, we took advantage of transgenic mice that express green fluorescent protein (GFP) in microglia and *in vivo* two-photon imaging to investigate the survival of endogenous microglia and the infiltration, repopulation and differentiation of host-derived microglia in an embryonic cortical tissue transplantation model. A parabiosis model was used to determine the fate and origin of both endogenous and host-derived microglia after successful transplantation. Our data demonstrated that vast majority of endogenous microglia in embryonic cortical tissue disappeared shortly after transplantation. Host-derived microglia infiltrated into the graft and restored the microglial population in the transplanted tissue, and local resident microglia in host brain parenchyma was the main source of colonized microglia.

## Results

### Endogenous microglia of the grafted tissue were lost rapidly after transplantation

**Figure 1:**
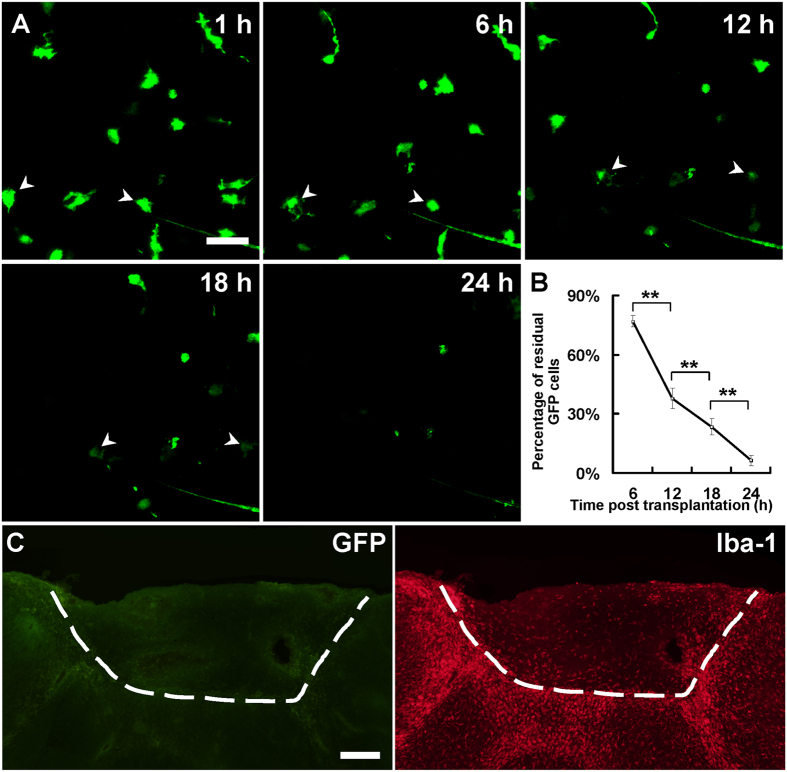
Loss of endogenous microglia in grafted tissue. (**A**) *In vivo* two-photon time-lapse imaging of GFP^+^ microglia in grafted tissue at 1, 6, 12, 18 and 24 h after transplantation. Most GFP^+^ cells were lost within 24 h after transplantation. (**B**) Percentage of residual GFP^+^ cells in grafted area at 6, 12, 18 and 24 h after transplantation; compared with the number of cells at 1 h. N = 2–3 sections/mouse, 3–6 mice/group, **P < *0.01*. (**C**) An example showing GFP^+^ endogenous microglia disappeared in grafted tissue at 7 d after transplantation in most cases. Note the host animal was wild-type C57BL/6 mouse and the transplanted tissue was from a CX3CR1^GFP/+^ mouse. The white dash line shows the boundary between donor and recipient tissue. Scale bar, 25 μm (**A**); 200 μm (**C**).

We first established a transplantation model by transplanting embryonic cortical tissue from E14~E15 YFP H-line fetuses into wild-type adult mouse brains (Supplementary Fig. 1A,C). Consistent to the results of a previous study^7^, we found that grafted neurons can survive and differentiate in the host brains (Supplementary Fig. 2). Importantly, we found the somata of the grafted neurons located only in grafted tissue, but their axons and dendrites projected into the host tissue (Supplementary Fig. 2D–F). In addition, we also performed experiments to transplant embryonic cortical tissue from wild type pregnant mice to adult YFP H-line mice, and we did not find any soma of the YFP-marked host neurons in grafted tissue (Supplementary Fig. 2C), suggesting neurons in grafted tissue are not from the host brains. To investigate whether the endogenous microglia of the grafted embryonic tissue could survive and repopulate in host brain, we transplanted embryonic cortical tissue from E14~E15 CX3CR1^GFP/+^ fetus into the lesion cavity of the cortex of an adult wild-type mouse. Two-photon imaging through the open-skull windows was used to examine microglial dynamics in the transplants (Supplementary Fig. 1A,B). We observed drastic atrophy of the endogenous microglia within hours after transplantation and the number of GFP^+^ cells decreased gradually in grafted tissue (Fig. 1A). There were only 37.7 ± 5.2% and 6.3 ± 2.7% of GFP^+^ cells left at 12 h and 24 h after transplantation, respectively (Fig. 1B), and repeated imaging showed that no GFP^+^ cells was found in the transplants at 36 h after transplantation (Supplementary Fig. 3A). To reduce the possible impact of repeated imaging on microglial survival, we imaged the transplanted tissues only once at 36 hours after grafting, and still no GFP^+^ cells was found in the graft (Supplementary Fig. 3B). TUNEL staining of the grafted tissue at 6 h after transplantation showed that some of the grafted microglia were TUNEL-positive (Supplementary Fig. 4), suggesting the grafted microglia can undergo apoptotic cell death.

To further confirm the loss of endogenous microglia and determine if any microglia existed in the transplants, brain sections of the grafted tissue from 7 days to 60 days after transplantation were harvested and stained with IBA-1 antibody. We found that abundant of IBA-1 positive microglia were distributed in grafted tissue at 7 d and 60 d (Fig. 1C, Supplementary Fig. 5A). However, we failed to find any GFP^+^ cells in the grafted tissues of most of the animals (13 out of 15 mice, Fig. 1C). Exceptionally, very few sparsely distributed GFP^+^ cells were found survived in the transplants in 2 animals (2 out of 15 mice, Supplementary Fig. 5A). These GFP^+^ cells only constitute about 0.1% of total IBA-1 positive cells, but they exhibited a similar morphology to neighboring ramified microglia (Supplementary Fig. 5B). Together, these results demonstrated that vast majority of the donor-derived microglia in the grafted tissue failed to survive in host brain after transplantation.

### Microglia survived in the grafted tissue are colonizers from the host

**Figure 2:**
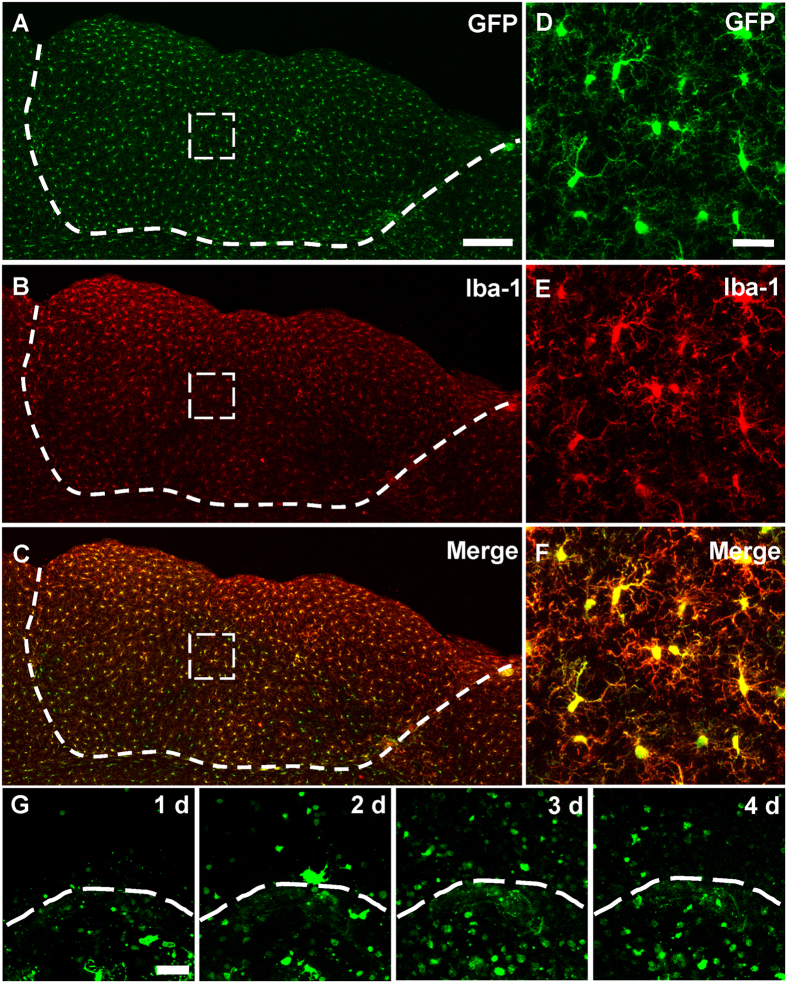
Colonization of host-derived microglia in grafted tissue. (**A**) GFP^+^ host-derived microglia (green) existed homogeneously in grafted tissue at 60 d after transplantation. (**B**) IBA-1 antibody staining (red) showing microglia in transplanted cortical tissue. (**C**) Merge of channel (**A**,**B**) showing that the majority of GFP^+^ microglia were also IBA-1 positive. N = 4–5 sections/mouse, 3 mice. (**D–F**) Representative higher magnification image of boxed-region in (**A**–**C**). (**G**) Images taken through an open-skull window showing the migration of microglia from CX3CR1^GFP/+^ mouse cortex to wild-type embryonic cortical graft at 1, 2, 3, and 4 days after transplantation. Only sporadic GFP^+^ microglia crossed the border line at 1 day after transplantation, and the cell density of GFP-positive cells increased in the grafted tissue from 1–3 d, then decreased at 4 d. Note the host animal was CX3CR1^GFP/+^ mouse and the transplanted tissue was from a wild-type C57BL/6 mouse. The white dash line shows the boundary between donor and recipient tissue. Scale bar, 200 μm (**A–C**); 25 μm (**D**–**F**); 50 μm (**G**).

The distribution of IBA-1 positive but GFP-negative microglia in the transplants suggested that host microglia can infiltrate the donor tissue. To confirm this, embryonic cortical tissue from wild-type fetus was grafted into the lesion cavity of adult CX3CR1^GFP/+^ mouse (Supplementary Fig. 1A,C). At 60 days after transplantation, we found that GFP^+^ cells were ubiquitously distributed in the graft (Fig. 2A). IBA-1 staining indicated that 98.9 ± 0.3% of the GFP^+^ cells in the graft were IBA-1-positive and 99.6 ± 0.3% of the IBA-1 positive cells were GFP^+^ (Fig. 2A–F). Theses data suggested that host brain controlled the great majority of microglial population in grafted tissue.

**Figure 3:**
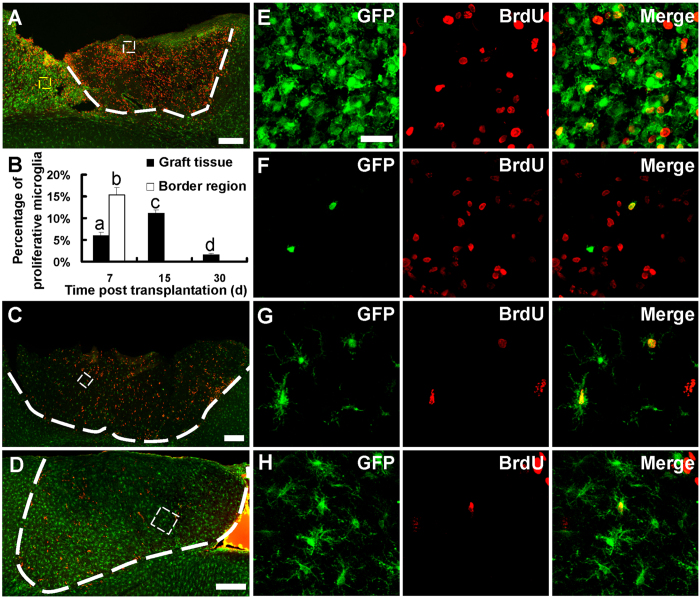
Proliferation of host-derived microglia at different time points after transplantation. (**A**,**C**,**D**) Confocal images showing proliferative microglia (BrdU-positive and GFP-positive) at 7 d, 15 d and 30 d after transplantation. Note the graft was from a wild-type mouse and host animal was a CX3CR1^GFP/+^ mouse. White dash line shows the boundary of grafted tissue. (**B**) Percentage of proliferative microglia in grafted tissue and host part of border region at different time points. N = 3 sections/mouse, 3–4 mice/group. a, b, c, d indicating significant differences between the two groups. *P* < 0.01. (**E**) Higher magnification image of the yellow box region in (**A**) showing BrdU-positive microglia in host part of the border region next to grafted tissue at 7 d. (**F–H**) Higher magnification image of the white box region in (**A**,**C**,**D**) showing BrdU-positive microglia in grafted tissue at 7 d, 15 d and 30 d after transplantation. Scale bar, 200 μm (**A**,**C**,**D**); 25 μm (**E–H**).

We next examined the dynamics of microglial infiltration. Daily imaging through an open-skull window showed that GFP-marked cells from the host brain invaded into grafted tissue within 1 d after transplantation. The number of GFP-positive cells increased in the grafted tissue from 1–3 d, and then decreased at 4 d (Fig. 2G; cell densities were 128.0 ± 34.6/mm^2^, 296.7 ± 42.6/mm^2^, 770.5 ± 110.0/mm^2^ and 446.6 ± 41.0/mm^2+^ for 1 d, 2 d, 3 d and 4 d, respectively). Immunostaining of the fixed tissue indicated that microglia were activated in the host tissue near the border region during the first week after transplantation (Fig. 3A). We found that 15.3 ± 1.7% of the activated microglia were proliferative (both GFP-positive and BrdU-positive) in the host tissue near the border region (Fig. 3B,E) at 7 d after transplantation. However, no proliferative microglia was found in the host tissue along the border region (tissue surrounding the graft) at 15 d and later time points (Supplementary Fig. 6A,B). These results further suggested that microglia from host brain infiltrated into the donor tissue at early stage after transplantation.

### Infiltrated CX3CR1^GFP/+^ cells restored the microglial population in grafted tissue

**Figure 4:**
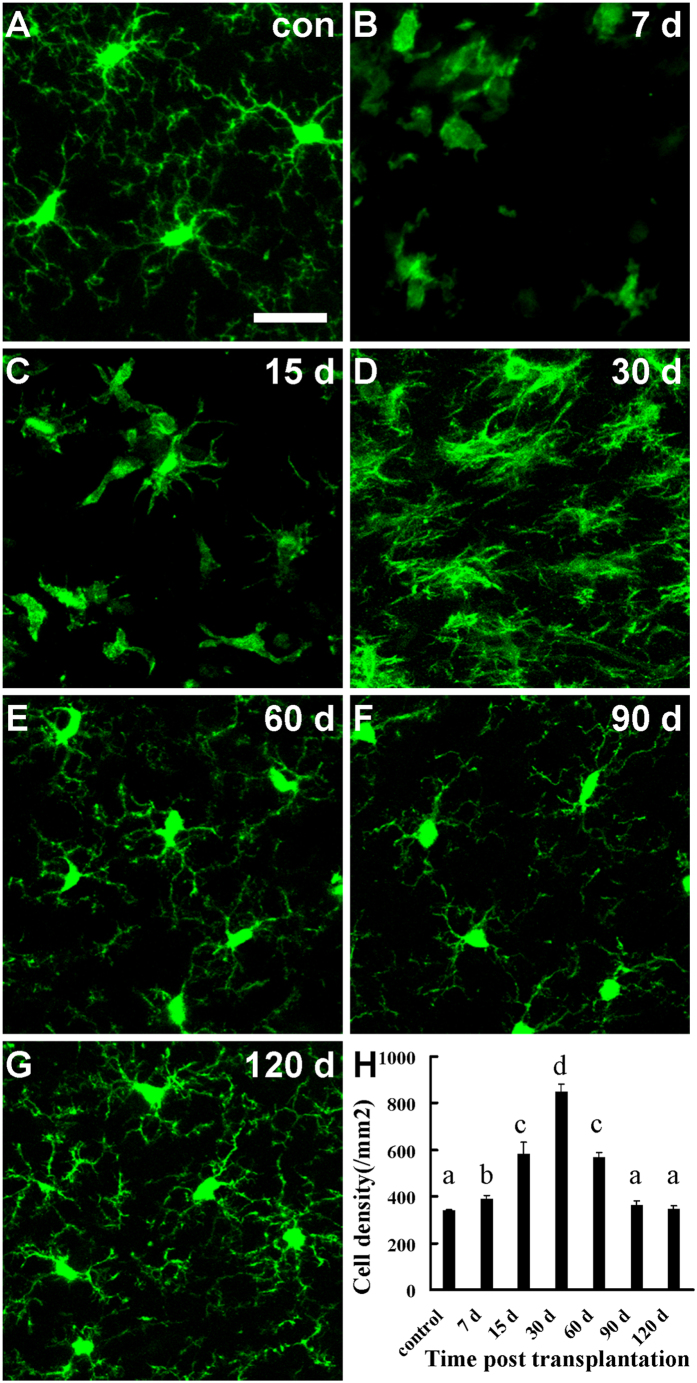
Changes in density and transition in morphology of host-derived microglia in grafted tissue following transplantation. (**A**) Resting microglia in healthy CX3CR1^GFP/+^ mouse exhibited ramified structures. (**B–G**) Morphological changes of intruding microglia in grafted tissue at 7 d (**B**) 15 d (**C**) 30 d (**D**) 60 d (**E**) 90 d (**F**) and 120 d (**G**) after transplantation. Amoeboid microglia was the major phenotype at 7 d (**B**), microglia extended thick branches at 15 d (**C**), developed dense dendrites at 30 d (**D**) and restored gradually to ramified phenotype after 60 d following transplantation. (**H**) Quantification of the density of microglia in the graft cortical tissue at different time points following transplantation. N = 5–6 sections/mouse, 3–5 mice/group. a, b, c, and d indicating significant differences between the two groups. *P* < 0.01. Scale bar, 25 μm (**A–G**).

To better understand the migration and transition of host microglia, we evaluated the density and morphological transformation of microglia in transplanted tissue using fixed brain slices harvested from 7 d to 120 d after transplantation. Microglia in the graft had already reached a density higher than that in control animals at 7 d after transplantation (Fig. 4A,B and H; 338.6 ± 6.4/mm^2^ for control, 389.8 ± 15.5/mm^2^ for 7 d; n = 5 mice). Among these cells, 6.0 ± 0.7% of them were BrdU positive (Fig. 3F). The number of microglia increased gradually and reached a peak density at 30 d after transplantation (Fig. 4D,H; 848.0 ± 35.7/mm^2^, n = 5 mice), and then decreased to control level at 90 d and 120 d (Fig. 4F–H; 364.8 ± 16.5/mm^2^ for 90 d, 346.9 ± 12.8/mm^2^ for 120 d; n = 3 mice). Consistent to the density increase in microglia, we found that 11.1 ± 0.7% and 1.6 ± 0.3% of microglia were proliferative at 15 d and 30 d after transplantation respectively (Fig. 3G,H), but there was no BrdU positive microglia present in the graft at the subsequent time points after microglia reached a peak density (Supplementary Fig. 6C,D). Colonized microglia in the graft underwent a drastic morphological transition from amoeboid phenotype to ramified phenotype after transplantation (Fig. 4B–G). During the first two weeks after transplantation, amoeboid microglia with thick twigs was the predominant phenotype (Fig. 4C). Microglia transformed into “bushy microglia” with dense dendrites at 30 d^30^,^31^ (Fig. 4D) and restored to resting ramified morphology from 60 d to 120 d after transplantation (Fig. 4E–G).

### Parenchymal microglia in host brain is the main source of the colonized microglia

**Figure 5:**
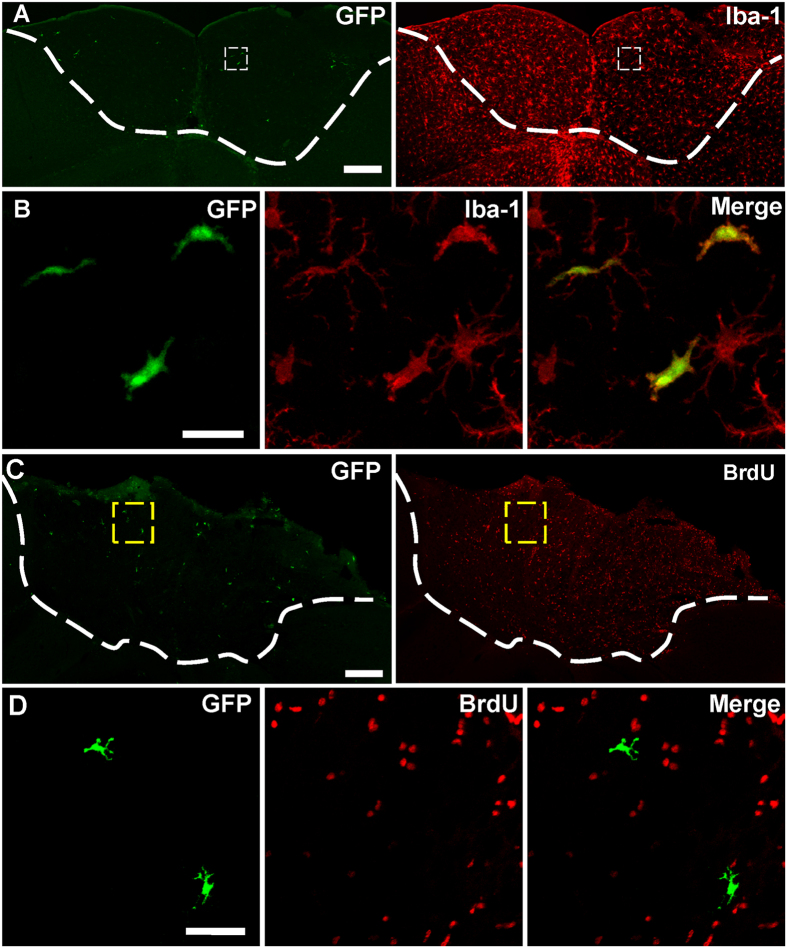
Infiltration of CX3CR1^GFP/+^ circulating cells into grafted tissue. (**A**) Distribution of CX3CR1^GFP/+^ infiltrating cells (green) and IBA-1 positive cells (red) in grafted tissue at 15 d after transplantation. (**B**) Higher magnification image of the boxed region in (**A**). Note that all of the GFP^+^ cells were IBA-1 positive but most of IBA-1 positive cells were not GFP^+^. N = 3–4 sections/mouse, 3 mice. (**C**) Distribution of CX3CR1^GFP/+^ infiltrating cells (green) and BrdU-positive cells (red) in grafted tissue at 15 d after transplantation. (**D**) Higher magnification image of the boxed region in (**C**), showing the CX3CR1^GFP/+^ infiltrating cells (green) were not BrdU-positive (red) in grafted tissue. N = 3 sections/mouse, 3 mice. Parabiotic surgery was performed between a wild-type mouse and a heterozygous CX3CR1^GFP/+^ mouse. Embryonic cortical tissue from a wild-type fetus was transplanted into the wild-type animal of the parabiotic pairs. The white dash line shows the boundary between donor and recipient tissue. Scale bar, 200 μm (**A**,**C**); 25 μm (**B**,**D**).

Above data revealed that microglia in the grafted tissue were from host brain. However, it remains unclear whether infiltrating microglia are from peripheral circulating cells or resident microglial pool in brain parenchyma. To investigate the origin of colonized microglia, parabiotic surgery was first performed between a wild-type mouse and a CX3CR1^GFP/+^ mouse, and embryonic cortical tissue from C57BL/6 fetus was grafted to the wild-type mouse of the parabiotic pairs (Supplementary Fig. 1D). We observed only a small number of CX3CR1^GFP/+^ cells distributed in grafted tissue (Fig. 5A). All of these infiltrating cells were IBA-1 positive (Fig. 5B), but these infiltrating cells (GFP-positive and IBA-1 positive) accounted for only 3.8 ± 1.2% of the total IBA-1 positive cells in the graft. BrdU assay showed that none of these infiltrating cells were BrdU-positive (Fig. 5C,D), suggesting infiltrating cells from bloodstream do not proliferate. Thus, circulating cells from bloodstream do not contribute to proliferative microglia and parenchymal microglia of the host brain is the main source of the microglial population in the graft tissue.

## Discussion

Our data suggest that parenchymal microglia from the host brain is the main source of microglial population in grafted cortical tissue. These results revealed an interactive relationship between the grafted tissue and the host brain after transplantation. The host brain benefited from grafted neurons in promoting cortical circuit reconstruction and function recovery. Meanwhile, infiltrating cells from host brain restored the microglial population of grafted tissue and may play an important role in monitoring and supporting the microenvironment of grafted tissue.

Repairing the injured neural network by transplantation of embryonic cortical tissue has provided a potential therapy in the treatment of brain injuries and diseases^7^,^9^,^32^. Grafted neurons can differentiate and project efferent myelinated fibers to appropriate cortical and subcortical targets^2^,^4^,^7^. Consistently, we found that donor-derived neurons can survive and integrate successfully into the host brain. Unlike the graft neurons, our results revealed that vast majority of the microglia from graft tissue disappeared after the transplantation. The survival of neurons but not microglia in grafted tissue indicates that microglia are more vulnerable than neurons in host environment. An *in vitro* model suggest that hypoxia/reperfusion can induce apoptosis of microglia, but not neurons under certain conndition^33^. Microglia exhibit various degree of viability in different experimental models. Microglia can maintain their population in cell cultures^34^,^35^ and slice cultures^36^,^37^,^38^, but are vulnerable to ischemia and nutrition deprivation^39^,^40^,^41^,^42^. Some studies have shown that grafted microglia can survive in host region in cell suspension transplantations^43^,^44^, but another cell suspension transplantation study indicate that grafted microglia are negligible to the overall microglial population within the graft area^45^. We observed endogenous microglia of the graft underwent a rapid atrophy and only in two rare cases that few endogenous CX3CR1^GFP/+^ cells survived in the transplants, suggesting that the traumatic environment used in our study doesn’t support the survival of embryonic microglia. The few CX3CR1^GFP/+^ cells observed in two animals in our study suggested that microglia from embryonic transplants can survive in the host brains if properly treated. Nevertheless, the rapid loss of endogenous microglia suggested that microglia from the embryonic tissue may not play an essential role in graft survival and differentiation.

When other different types of cells, such as mesenchymal stem cells, were transplanted into CNS, the transplants can cause strong immune response within the host^46^,^47^,^48^. During this process, massive activated microglia from host were observed to surround and invade into the grafted area^46^,^47^. Similarly, increasing number of activated CX3CR1^GFP/+^ cells from host were found to invade the grafted tissue in a few days after transplantation in the present study. In addition, these infiltrating cells resulted in reestablishing the normal microglial density of grafted tissue, and accounted for overwhelming majority of the population of the IBA-1 positive cells in the grafted tissue. These results indicated that invaded microglia from host dominated and restored the microglial population of grafted tissue, which had lost this important cell subset rapidly after transplantation.

Combination of transplantation surgery and parabiosis model demonstrated that the main source of colonized microglia in the transplant were parenchymal microglia of the host brain. Previous studies suggest that local expansion of reactive microglia, infiltration of circulating cells, or proliferation of latent progenitors in the CNS can contribute to activated microglia under various pathological conditions^26^,^27^,^49^,^50^. Consistent with our previous study in a stroke model^26^, here we observed that circulating cells infiltrated into the transplant, but the number of infiltrating cells constitutes only a small portion of IBA1-labeled cells. Importantly, these infiltrating cells do not proliferate and may not contribute to the sustained microglial population.

Despite the continuous increase in microglia density in the transplant during the first month after transplantation, we found proliferative microglia existed only within the first weeks in the host tissue near the border region, suggesting microglial infiltration occurred only during the early stage after transplantation. Infiltrated microglia were proliferative within the graft before they reached peak density. We observed that colonized microglia underwent a progressive transformation before they developed into highly ramified structures. These morphological and density changes largely recapitulate the developmental process of microglia during embryonic and postnatal periods^51^,^52^,^53^. Microglia are inflammatory cells in the CNS^11^,^20^,^54^ and one of the key players for circuit rewiring and synapse plasticity^16^,^17^,^22^,^53^. Their roles in the survival and differentiation of grafted embryonic tissue remain to be determined.

## Methods

### Animals

CX3CR1^GFP/+^, YFP H-line transgenic mice and C57BL/6 wild-type mice were used in this study. The animals were purchased from Jackson Laboratory and bred on a 12 h/12 h light/dark cycle with food and water ad libitum in the Laboratory Center for Medical Sciences, Lanzhou University. Mice aged 3–4 months of both sexes were used in this study. All experimental procedures and protocols in the study were in accordance with the ‘Guidance of the Care and Use of Laboratory Animals’ approved by Ethic Committee of Experimental Animals of Lanzhou University, China.

### Transplantation surgery

The transplanting procedure used in this study was modified from a method described previously^7^. Briefly, adult mice (as recipients) were anesthetized with an intraperitoneal injection of 20 mg/ml ketamine and 2 mg/ml xylazine. A piece of cranial bone of approximately 2 mm in diameter, in the skull of the recipient animal was removed by using dental drill at the position about 2.5 mm posterior to the Bregma and 2.5 mm lateral to the midline. Embryonic cortical tissue was dissected out from E14-15 d fetus cortex and cut into small fragments of about 1 mm^3^. When the embryonic cortical block was ready for use, a unilateral traumatic lesion, about 1 mm in diameter and depth, was made in the cortex of the recipient animal by using a dental drill. Care was taken to avoid making a through hole in cortex and keeping away from large vessels when drilling the hole. Immediately after the lesion, embryonic cortical graft was deposited into the lesion cavity. For brain harvest after transplantation, the removed cranial bone was put back to cover the exposed brain. Then, the separated skull was glued with the cranial bone by adhesives. After transplantation, the scalp was sutured and disinfected carefully, and animals were put back to the cages and housed for different time periods. For *in vivo* imaging, exposed brain was covered by a circular cover glass and the edge of the cover glass were sealed with dental acrylic as the methods previously reported^55^.

### Parabiosis

The parabiotic surgery was carried out following the procedure as previously reported^56^,^57^. A C57BL/6 (wild-type) mouse and a weight-matched heterozygous CX3CR1^GFP/+^ mouse were selected for the parabiosis surgery. Mice were anaesthetized by intraperitoneal injection with xylazine and ketamine, and the opposite flank of each mouse were shaved and sterilized with 75% ethanol followed by betadine solution. Then, a straight incision from the shoulder to the hip was made on the cleaned skins of each animal. Corresponding scapulas of each mouse were fixed together with non-absorbable 4-0 silk and the posterior muscles were sutured together by absorbable 5-0 chromic gut. Finally, the corresponding dorsal and ventral skin edges of each mouse were sutured with 4-0 silk. Pairs of mice were kept warm before completely recovered from anesthesia, and then they were put back to the cages and fed with food and water at 20 ± 2 °C room temperature. It took 14–15 days to establish sufficient anastomotic blood circulation and complete blood sharing between the pair of mice. Then, embryonic cortical tissue from C57BL/6 fetus was grafted to the wild-type mouse of the parabiotic pair.

### Immunohistochemistry

Mice were anesthetized intraperitoneally with a lethal dose of ethyl urethane and then transcardially perfused with 0.1 M PBS followed by 4% paraformaldehyde (PFA). The mouse brains were dissected out and soaked in 4% PFA for at least 36 hours at 4 °C. The fixed mouse brains were serially sectioned into 30 μm coronal slices on a vibrating microtome (TV1000S, Leica). For antibody staining, brain slices were pre-incubated with PBS-T (0.3% Twen-20 in 0.1 M PBS) for 0.5 h at room temperature (22 ± 2 °C). The slices were washed three times with PBS and then stained overnight in primary antibodies at 4 °C on a shaker. For primary antibodies, we used a rabbit antibody to IBA-1 as a microglia marker (1:500, Wako), mouse antibody to NeuN as a neuron marker (1:250; Millipre). Second antibodies were TRITC-conjugated goat anti-rabbit (1:100; ZSGB-BIO), FITC-conjugated goat anti-rabbit (1:100; ZSGB-BIO), and FITC-conjugated goat anti-mouse (1:100; ZSGB-BIO).

To mark proliferating cells, bromodeoxyuridine (BrdU, 50 mg/kg; Sigma) was injected intraperitoneally per 2 hours for four times one day before brain harvest. For BrdU staining, brain slices were rinsed in double distilled water, and treated in 2 M HCl at 37 °C for 30 min, then neutralized with 0.1 M borate buffer (PH 8.5) for 3 × 15 min and incubated overnight in rat anti-BrdU (1:500; AbD Serotec). Second antibodies were TRITC-conjugated goat anti-rat (1:100; ZSGB-BIO) and the sections were stained for 1.5 h at room temperature in the dark.

### *In vivo* two-photon and confocal microscopy imaging

Brain sections after fluorescent staining were imaged with UPlan SApo ×40/0.85 objective on a confocal laser scanning microscope (Olympus FV1000). Stacks (1024 × 1024 pixel arrays) were captured at 1 μm intervals in the Z dimension. *In vivo* two-photon imaging was performed as described previously^58^. Briefly, mice were deeply anesthetized by intraperitoneal injection of xylazine and ketamine. For stable imaging, the optical window was fixed to a custom-made steel plate. To excite GFP fluorescence, the mouse was fitted into a two-photon microscope and imaged with ×25 water-immersion objective (×25/1.05; Olympus) at laser of 890 nm.

### Data analysis

To calculate the disappearance rate of endogenous microglia, numbers of GFP^+^ cells at 6 h, 12 h, 18 h, 24 h after transplantation were compared with the number of GFP^+^ cells at 1 h after transplantation under the same imaging area. To compare the density of the microglia in grafted tissue at different time point, a random area in grafted tissue was selected to count the microglia number. To quantitate the percentage of proliferating microglia, three slices from each mouse brain were randomly chosen, and all of the microglia and BrdU-positive microglia in grafted tissue and host part of border region (accumulation area contain large number of both activated microglia and BrdU-positive cells near the grafted tissue in host brain) were counted. All areas are measured by using ImageJ software. Statistical analyses of all dates were done by using IBM SPSS Statistics 19 software (One way ANOVA for three or more groups, and unpaired t-test for two groups). All date are presented as mean ± SEM.

## Additional Information

**How to cite this article**: Wang, C. *et al.* Infiltrating cells from host brain restore the microglial population in grafted cortical tissue. *Sci. Rep.* **6**, 33080; doi: 10.1038/srep33080 (2016).

## Supplementary Material

Supplementary Information
