# Supplementary material for: Infiltrating cells from host brain restore the microglial population in grafted cortical tissue
Source: Sci Rep. 2016 Sep 12;6:33080. doi: 10.1038/srep33080 (PMC5018877; doi:10.1038/srep33080)
Supplement: Supplementary Information [file srep33080-s1.doc]

**Infiltrating cells from host brain restore the microglial population in grafted** **cortical tissue**

Cong Wang^1^, Sijue Tao^1^, Yukun Fang^1^, Jing Guo^1^, Lirui Zhu^1^, Shengxiang Zhang^1^

^1^Gansu Key Laboratory of Biomonitoring and Bioremediation for Environmental Pollution, School of Life Sciences, Lanzhou University, Lanzhou 730000, China


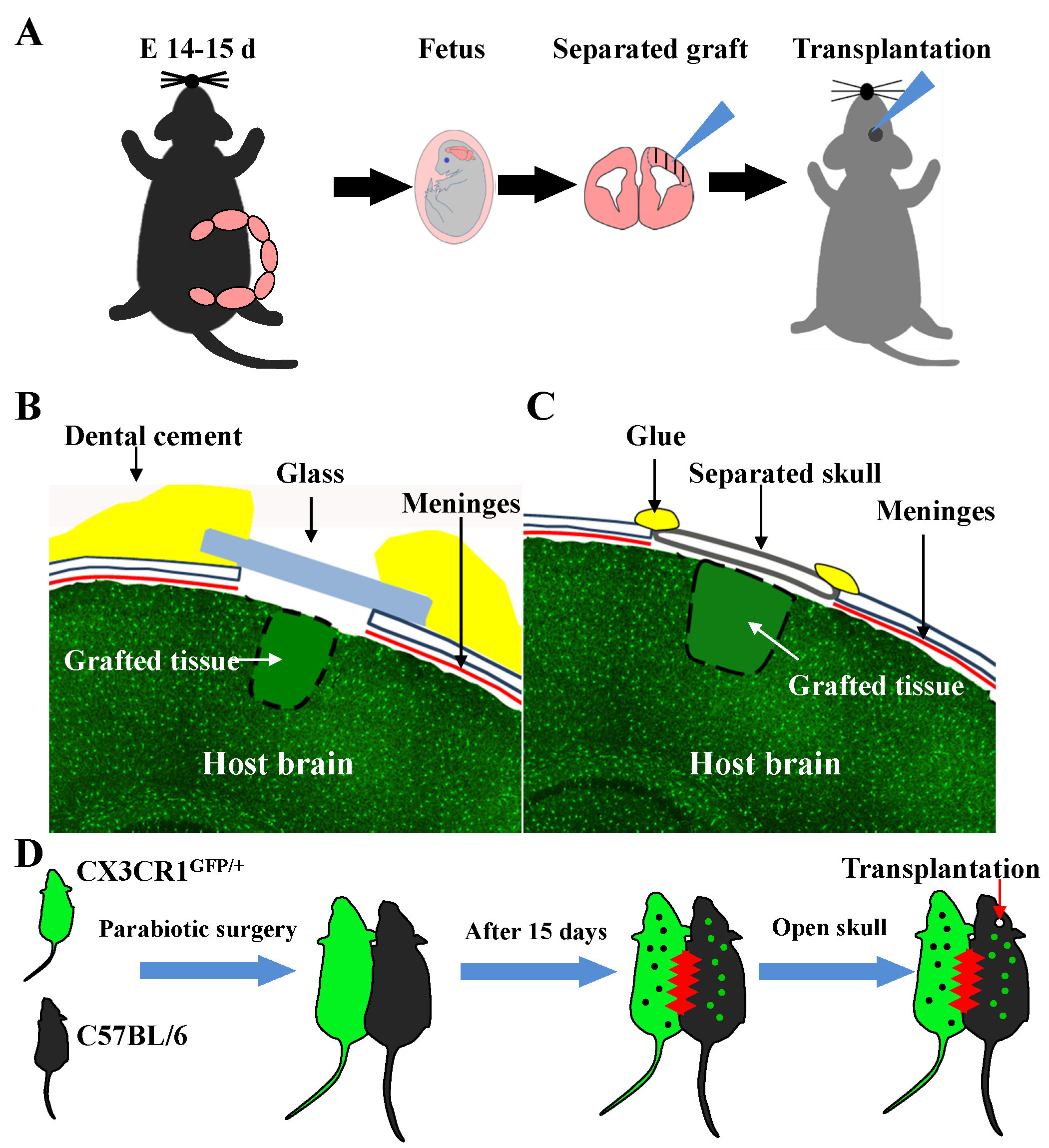


**Supplementary Figure 1 Diagram showing experimental methods used in this study.** (**A**) Surgery procedure for transplantation. Embryonic cortical tissue was taken from fetus of E14-15 pregnant mice, separated into fragments, and grafted into the lesion cavity in adult mouse cortex. (**B**) Open-skull window used to image the grafted tissue and host cortex after transplantation. After grafting, a circular glass was used to cover the open-skull window and immobilized with the cranium by dental cement. (**C**) For fixed tissue experiments, separated skull piece was placed back to cover the open skull after the grafting procedure shown in **A**. (**D**) Surgery procedure for parabiosis model: Wild-type mouse and CX3CR1^GFP/+^ mouse were fixed together by surgical operation for 15 days before transplantation. Note the host animal was a wild-type mouse.


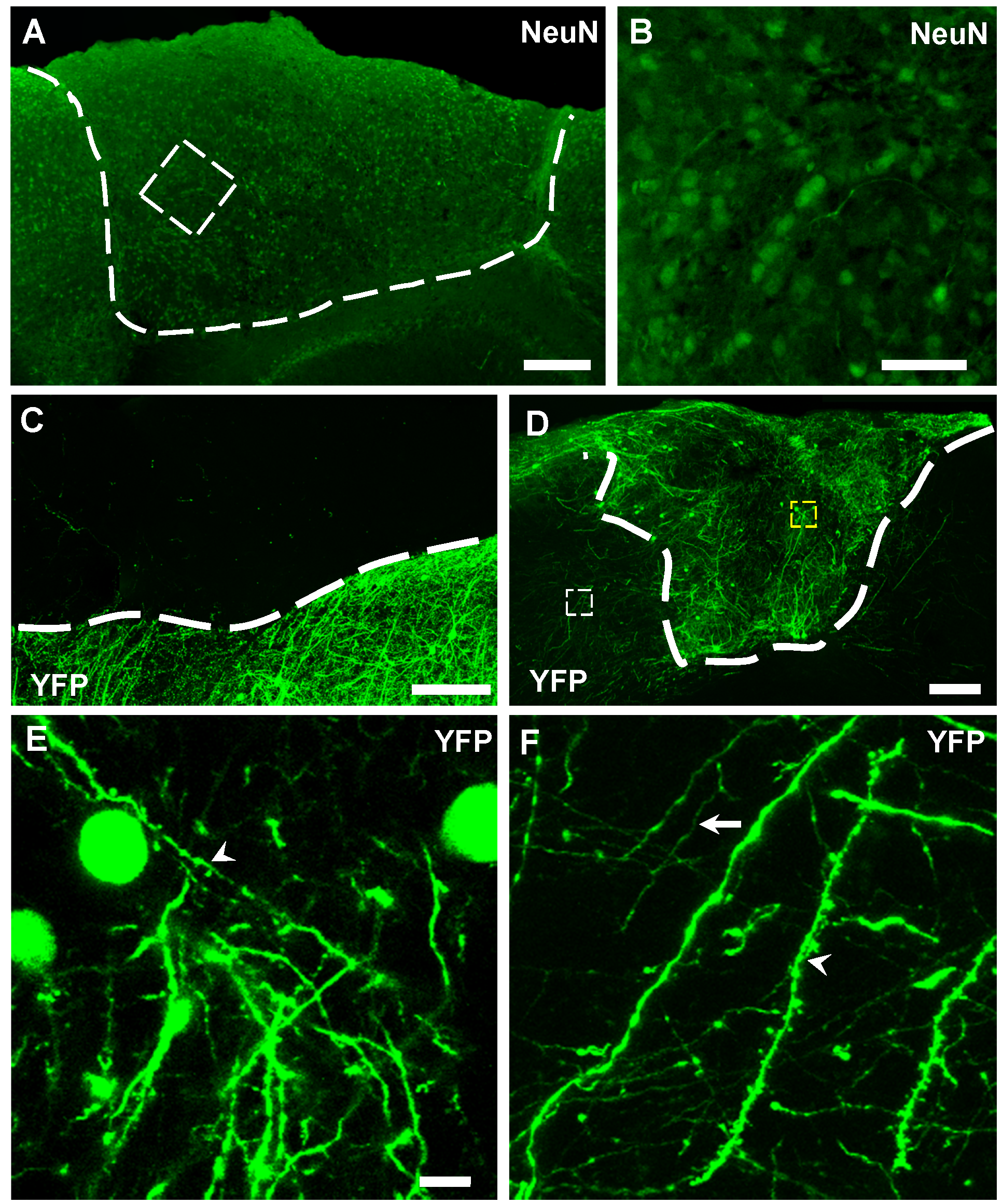


**Supplementary Figure 2 The viability of grafted neurons in host brain after transplantation. (A)** NeuN antibody staining (green) showing a large number of neurons in grafted tissue at 15 d after transplantation. Note the graft was from a wild-type fetus and the host animal was a wild-type mouse. **(B)** Magnified view of the white box region in **A.** (**C**) None of the soma of YFP^+^ neurons from host were found in grafted tissue when a graft from a wild-type fetus was transplanted into the host brain of an adult YFP H-line mouse at 60 d after transplantation. **(D)** The grafted neurons survived and differentiated well at 60 d after transplantation. The graft was from an YFP H-line fetus and the host animal was a wild-type mouse. Note the somata of grafted neurons located only in grafted tissue, but the axons and dendrites of these neurons projected into the host tissue **(E)** Higher magnification image of the yellow box region in **D** showing grafted neurons and their dendrite (arrowhead). **(F)** Higher magnification image of the white box region in **D** showing grafted neurons projected axon (arrow) and dendrite (arrowhead) into host brain. White dash line shows the boundary of grafted tissue. Scale bar, 200 μm (**A, C, D**); 50 μm (**B**); 25 μm (**E, F**).

**
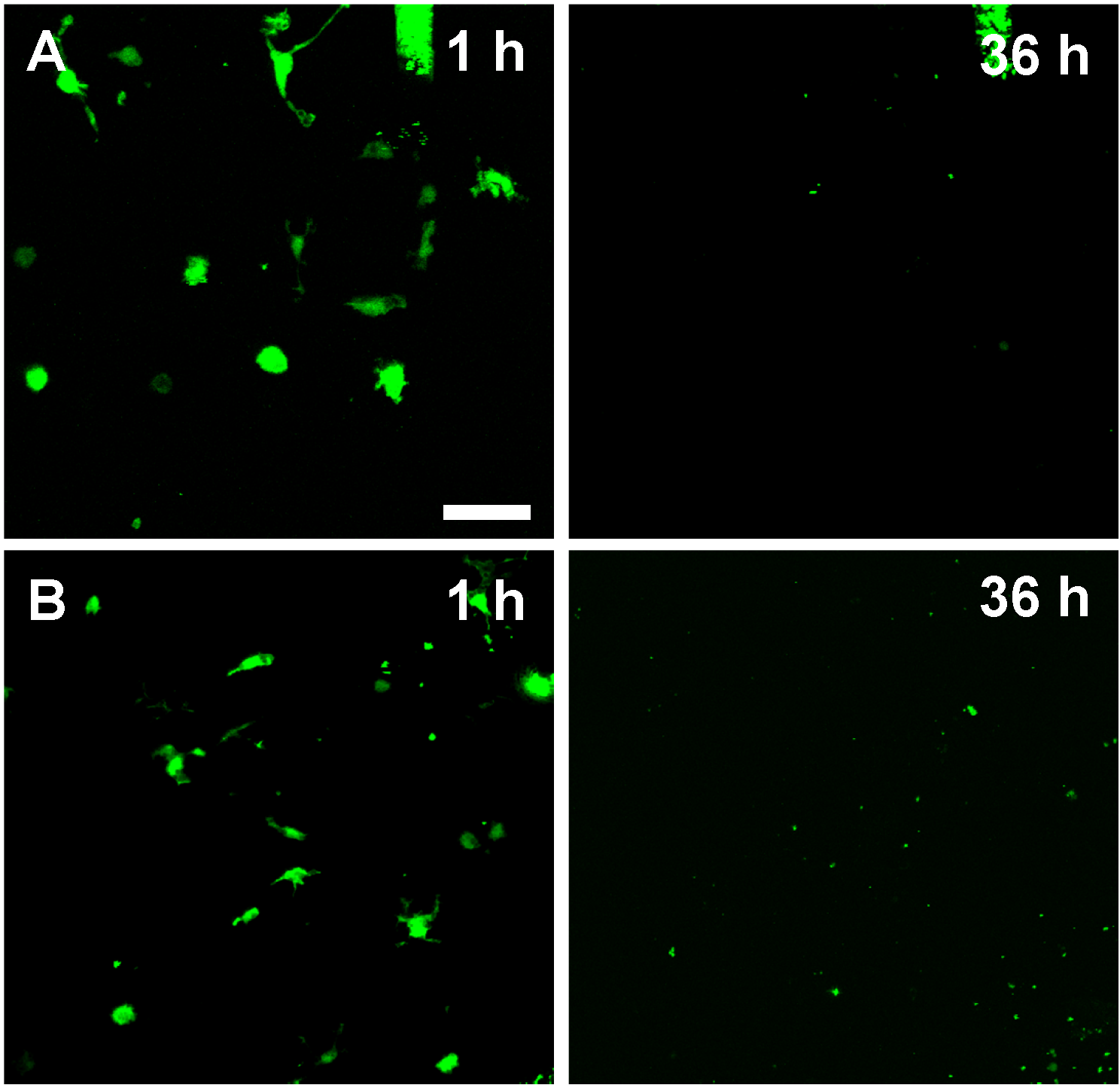
**

**Supplementary Figure 3 Loss of GFP^+^ cells in grafted tissue** (**A**) Repeated imaging of the GFP^+^ cells in grafted tissue from 1h to 36 h after transplantation showing no GFP^+^ cells left in the transplants. (**B**) In vivo two photon imaging of GFP^+^ microglia in grafted tissue only at 1 h and 36 h after transplantation showing no GFP^+^ cells was found in the transplants. Note the graft was from a CX3CR1^GFP/+^ fetus and the host animal was a wild-type mouse. N= 3 mice. Scale bar, 50 μm (**A, B**).

**
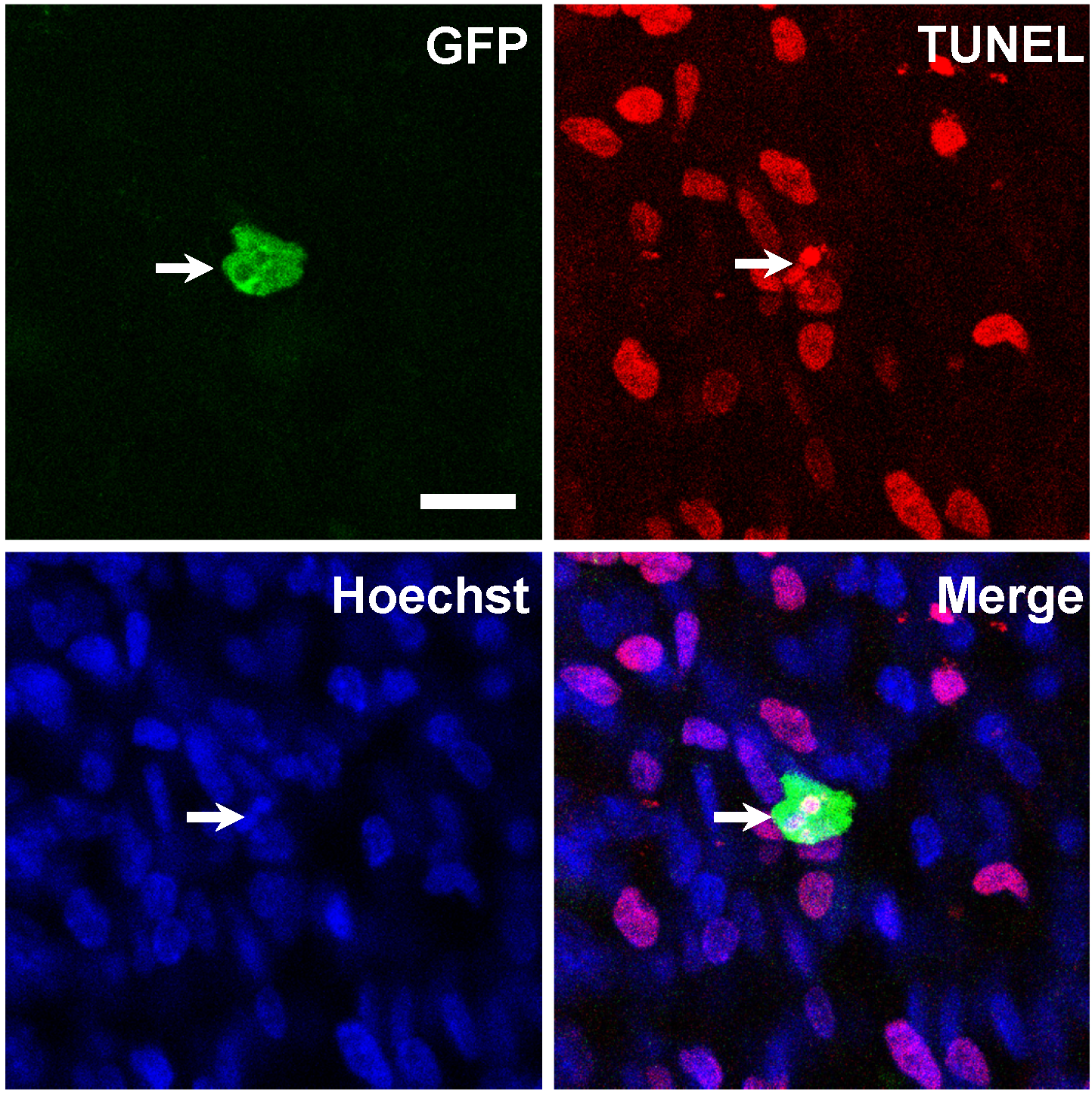
**

**Supplementary Figure 4 Apoptosis of grafted microglia after transplantation.** Arrow indicates a grafted microglia (GFP^+^) were TUNEL-positive at 6 h after the transplantation. Note the graft was from a CX3CR1^GFP/+^ fetus and the host animal was a wild-type mouse. Scale bar, 10 μm.

**
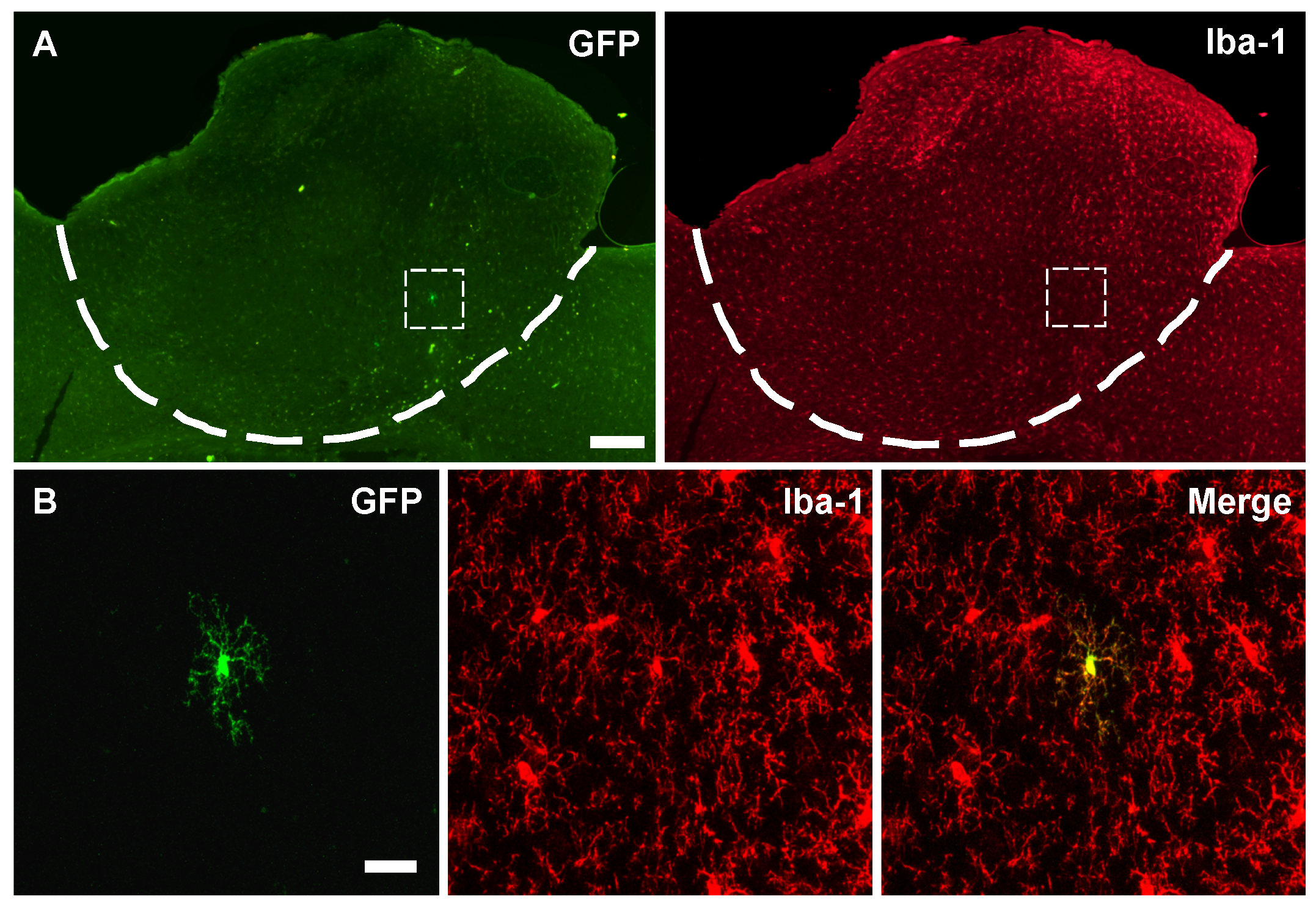
**

**Supplementary Figure 5 Survival of endogenous CX3CR1^GFP/+^ cells of grafted tissue after transplantation.** (**A**) A small number of endogenous CX3CR1^GFP/+^ cells (2 of 15 animals) were observed in grafted tissue at 60 d after transplantation, and the great majority of the IBA-1-labelled cells were GFP-negative. (**B**) Magnified view of the white box in **A**, showed that the GFP-positive cells were also IBA-1-positive. Note the host animal was a wild-type C57BL/6 mouse and the transplanted tissue was from a CX3CR1^GFP/+^ fetus. The white dash line shows the boundary between donor and recipient tissue. Scale bar, 200 μm (**A**); 25 μm (**B**).

**
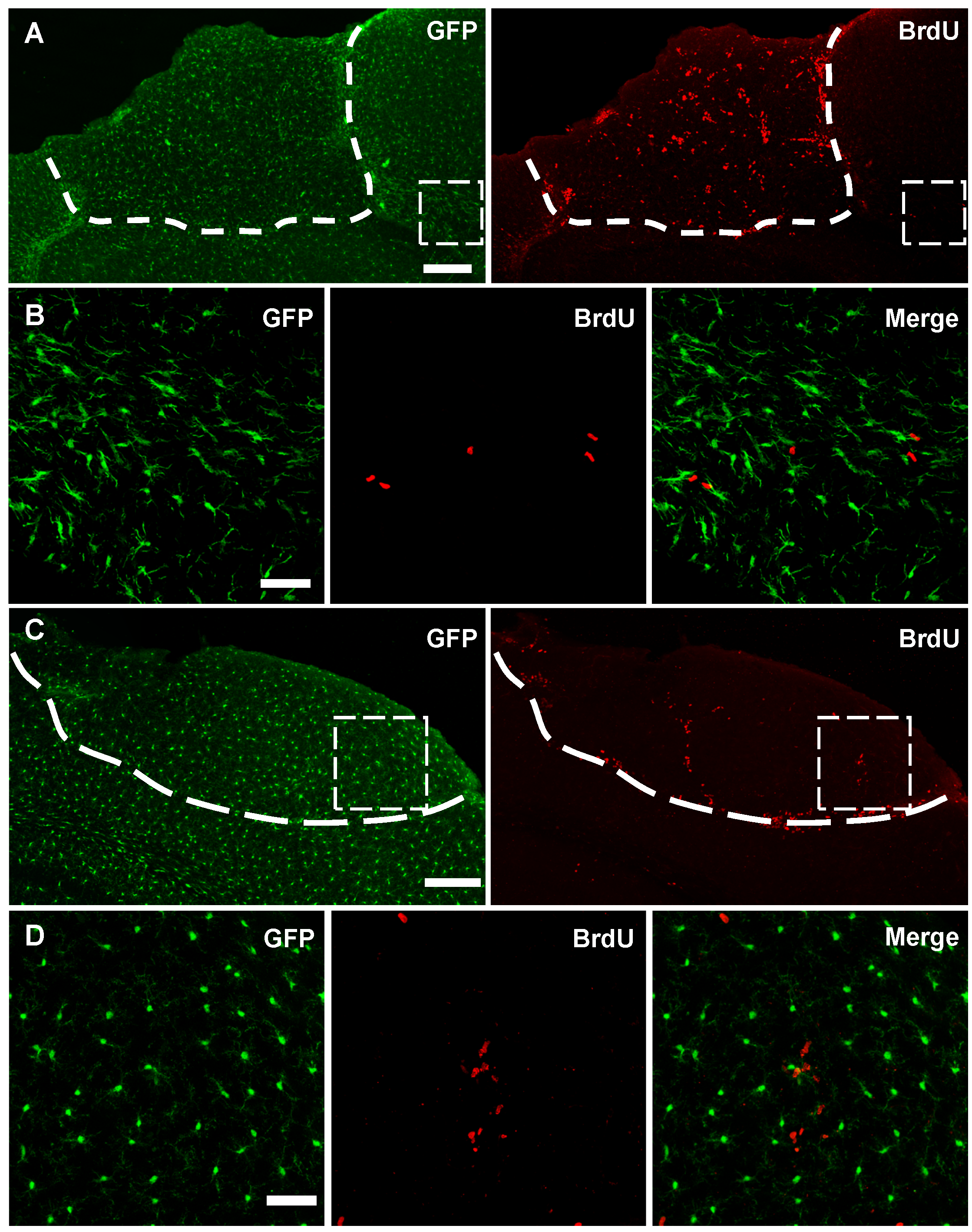
**

**Supplementary Figure 6 Proliferation of microglia in recipient tissue near the border region and grafted tissue at different time points after transplantation.** (**A, C**) Confocal images showing microglia (green) and proliferative cells (red) at 15 d and 45 d after transplantation, respectively. (**B**) Magnified view of the white box region in **A** showing no proliferative microglia located in the recipient tissue near the border region at 15 d after transplantation. (**D**) Magnified view of the white box region in **C,** showed no BrdU positive microglia in the grafted tissue at 45 d after transplantation. Note the graft was from a wild-type mouse and the host animal was a CX3CR1^GFP/+^ mouse. White dash line shows the boundary between grafted tissue and recipient tissue. N=3 mice. Scale bar, 200 μm (**A, C**); 50 μm (**B, D**).
